# Distribution and Antioxidant Efficiency of Resveratrol in Stripped Corn Oil Emulsions

**DOI:** 10.3390/antiox3020212

**Published:** 2014-04-04

**Authors:** Sonia Losada-Barreiro, Marlene Costa, Carlos Bravo-Díaz, Fátima Paiva-Martins

**Affiliations:** 1 Department of Physical Chemistry, Faculty of Chemistry, University of Vigo, Vigo 36200, Spain; E-Mail: cbravo@uvigo.es; 2 CIQ-UP, Department of Chemistry and Biochemistry, Faculty of Sciences, University of Porto, Porto 4169-007, Portugal; E-Mails: marlene_andreia_costa@hotmail.com (M.C.); mpmartin@fc.up.pt (F.P.-M.)

**Keywords:** resveratrol, corn oil-in-water emulsions, pseudophase model, antioxidant distribution, antioxidant activity

## Abstract

We investigated the effects of resveratrol (RES) on the oxidative stability of emulsions composed of stripped corn oil, acidic water and Tween 20 and determined its distribution in the intact emulsions by employing a well-established kinetic method. The distribution of RES is described by two partition constants, that between the oil-interfacial region, *P*_O_^I^, and that between the aqueous and interfacial region, *P*_W_^I^. The partition constants, *P*_O_^I^ and *P*_W_^I^, are obtained in the intact emulsions from the variations of the observed rate constant, *k*_obs_, for the reaction between the hydrophobic 4-hexadecylbenzenediazonium ion and RES with the emulsifier volume fraction, Ф_I_. The obtained *P*_O_^I^ and *P*_W_^I^ values are quite high, *P*_W_^I^ = 4374 and *P*_O_^I^ = 930, indicating that RES is primarily located in the interfacial region of the emulsions, %RES_I_ > 90% at Ф_I_ = 0.005, increasing up to 99% at Ф_I_ = 0.04. The oxidative stability of the corn oil emulsions was determined by measuring the formation of conjugated dienes at a given time in the absence and in the presence of RES. The addition of RES did not improve their oxidative stability in spite that more than 90% of RES is located in the interfacial region of the emulsion, because of the very low radical scavenging activity of RES.

## 1. Introduction

**Scheme 1:**
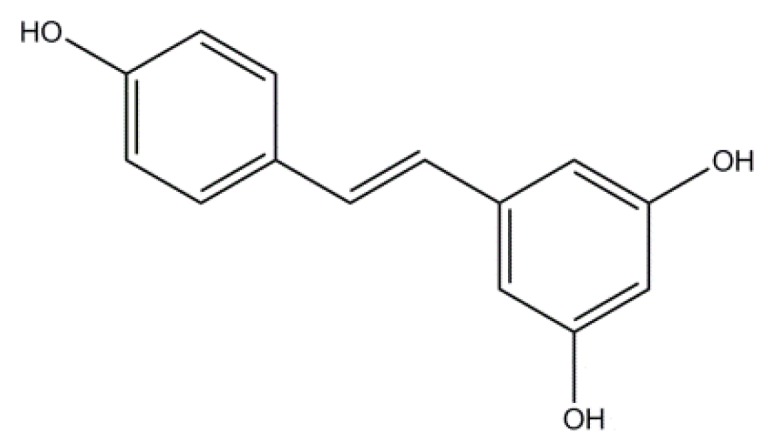
Chemical structure of *trans*-resveratrol (RES).

Resveratrol (3,5,4′-trihydroxystilbene) is a natural polyphenol found in grapes and grape-derived products, peanuts and other foods commonly found in the human diet [1]. It is also widely used in a broad range of medicinal applications [2,3] to inhibit or delay tissue oxidation [4], atherosclerotic [5], cancer processes [6], platelet aggregation [7], *etc.* Resveratrol shows *Z*-*E* isomerism. The *trans* (*E*) isomer, Scheme 1, is much more stable than the *Z*-isomer, and it is believed to be biologically more active, probably due to its planar conformation [8]. The interest of the food industry in resveratrol is related to its ability to inhibit lipid oxidation [1] in addition to its health-promoting benefits [3,8]; however, *trans*-resveratrol (RES) has low water solubility, leading to reduced oral bioavailability, and novel resveratrol formulations are being developed to overcome the development for therapeutic applications [8].

The antioxidant activity of RES has been investigated, and contradictory results have been reported. Wang *et al.* [9] showed that resveratrol is a better antioxidant than butylated hydroxytoluene (BHT) in pure lard (pork fat). Medina *et al.* [10] reported that resveratrol has an antioxidant activity similar to that of hydroxytyrosol in fish oil-in-water emulsions and fish muscle. In contrast, Filip *et al.* [11] reported that RES behaved as a weak antioxidant against the oxidation of sunflower and rapeseed oils and in water-in-oil emulsions (margarine). In attempting to increase and modulate the antioxidant efficiency of RES, Medina *et al.* [10] synthetized several acylated and glycosylated derivatives of RES of different hydrophobicity and found out that lipophilization of RES did not improve significantly or even reduced its antioxidant efficiency either in bulk fish oils or in cod liver oil-in-water emulsions. They hypothesized that the negligible antioxidant effect found in emulsions might be attributable to a differential incorporation of the RES derivatives in the oil, aqueous and interfacial regions of the emulsion droplets; however, they did not report on their distributions. 

**Scheme 2:**
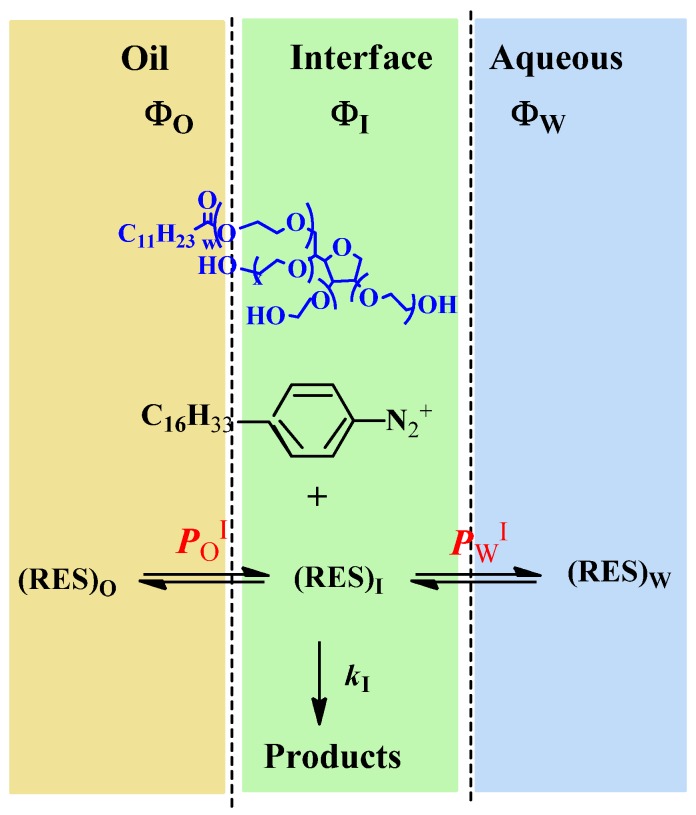
Basic illustration of a small portion of an emulsion showing the oil (O), aqueous (W) and interfacial (I) regions. Ф_I_, Ф_W_ and Ф_O_ indicate the volume fraction of each region (Ф_region_ = *V*_region_/*V*_Total_), and *P*_W_^I^ and *P*_O_^I^ are the partition constants describing the distribution of an antioxidant (here, RES); and, k_I_ is the interfacial rate constant of the reaction between the 4-hexadecybenzenediazonium ions (16-ArN_2_^+^) and the antioxidant.

Determining the distributions of antioxidants (AOs) in emulsified systems is complex, because of the physical impossibility of separating the interfacial region from the oil or aqueous regions [12,13,14,15,16]. In binary oil-water systems and in the absence of emulsifiers, the distribution of a molecule (e.g., an AO) can be determined by measuring the concentrations in the oil and water regions with suitable analytical techniques [17], and the ratio of concentrations yields its partition constant between the oil and water, *P*_W_^O^, Equation (1), where *V*_W_ and *V*_O_ are the volumes of the aqueous and oil phases, respectively, and parentheses, (), denote the concentration in mol/L of the volume of a particular region [18]. However, upon the addition of emulsifiers to prepare emulsions, a new region is created between the oil and aqueous regions and AOs distribute between the oil, aqueous and interfacial regions, Scheme 2 [19]. Two partition constants are required to describe the distribution of an AO, one between the aqueous-interfacial (*P*_W_^I^) and one between the oil-interfacial (*P*_O_^I^) regions, Equations (2) and (3), respectively. These partition constants cannot be measured independently by isolating and analyzing the concentration of AO in each region, because breaking the emulsion will break the equilibria and bias the results. Thus, the partition constants, *P*_O_^I^ and *P*_W_^I^, must be determined in the intact emulsions [12,13,15,16,19].


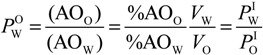
(1)


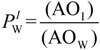
(2)


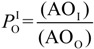
(3)

We have developed a kinetic method, Scheme 2, to assess the distribution of AOs in the intact emulsions [13,14,16,19]. The method exploits the reaction between 4-hexadecylbenzenediazonium ions, 16-ArN_2_^+^ and the antioxidants. In recent work, we showed [12,13,14] that the fraction of antioxidants in the interfacial region of emulsions do not correlate with their hydrophobicity, for instance, the fraction of hydrophilic antioxidants, such as gallic or caffeic acid in the interfacial region of emulsions is higher than that of hydrophobic antioxidants, such as α-tocopherol or lauryl gallate. Thus, the distribution of AOs in emulsions cannot be predicted solely on the basis of their hydrophobicity [12,13], and here, we have employed our methodology to investigate the effects of emulsifier concentration on the distribution of RES in stripped corn oil emulsions. We also determined its antioxidant efficiency in order to contribute to understanding the complex relationships between antioxidant distributions and their efficiencies in emulsified systems and improve the quality of lipid-based products. 

### Relationships between the Observed Rate Constant, k_obs_, and the Partition Constants, P_W_^I^ and P_O_^I^: The Pseudophase Kinetic Model

The conceptual basis of the method has been published [12,13,19], and only a brief summary is given here. The two basic assumptions are: (1) the distribution of reactants (in this case, RES) between regions in emulsions are in dynamic equilibrium, that is, the rates of transport of the reactants between regions of the emulsions are much faster than those of the chemical reactions (e.g., that between RES and 16-ArN_2_^+^); (2) the distribution of RES (and that of any other antioxidant) between the oil, water and interfacial regions depends on its relative solubility in each region and not on the size and shape of the droplets in the emulsions or the type of emulsion (oil-in water or water-in-oil).

The reaction between 16-ArN_2_^+^ and RES in each region of an emulsion is the product of the second-order rate constant and the concentration of each reactant in that region in moles per liter of region volume [19]. 16-ArN_2_^+^ has a long hydrophobic alkyl chain and a cationic headgroup and is both water and oil insoluble. Its reactive-N_2_^+^ group is located in the interfacial region, where it reacts with RES, as illustrated in Scheme 2, and its concentration in the oil and water regions can be considered negligible. Under pseudo-first order conditions (being [RES] much higher than [16-ArN_2_^+^]), the observed rate, *k*_obs_, is given by Equation (4) [19].


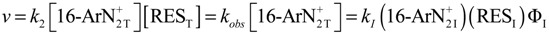
(4)

In Equation (4), *k*_2_ and *k*_I_ are the observed second rate constant and the second order rate constant in the interfacial region, respectively; *k_obs_* is the observed overall rate; square brackets, [], denote the concentration in mol/L of the total emulsion volume; the subscript T stands for total; parentheses, (), denote the concentration in mol/L of the volume of a particular region; subscript I stands for the interfacial region; and Ф_I_ is the emulsifier volume fraction defined as the ratio of the volume of emulsifier divided by the total volume of the emulsion (Ф_I_ = *V*_surf_/*V*_Total_). Bearing in mind Equations (2)–(4) and the relevant mass balance equations, Equation (5) can be derived [19,20]. Equation 5 describes the dependence of *k*_obs_ on both the concentration (*P*_W_^I^, *P*_O_^I^) and the medium (*k*_I_) and predicts that: (1) *k*_obs_ should decrease with increasing Ф_I_ at constant [RES]; Ф_W_ and Ф_O_ and (2) plots of 1/k_obs_ *vs*. Ф_I_, Equation (9), should be linear with a positive intercept.


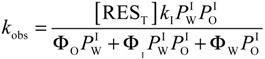
(5)


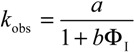
(6)


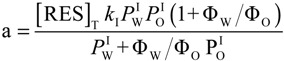
(7)


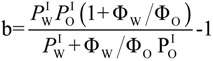
(8)


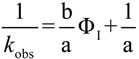
(9)

Equation (6) has the same form as Equation (5), where the parameters, *a* and *b*, are given by Equations (7) and (8), respectively. They can be determined by linear least squares fitting of the (1/*k_obs_*, Ф_I_) pairs of data. The partition constants, *P*_O_^I^ and *P*_W_^I^, can be obtained by two different methods [19,20]. Here, we determined their value by combining the *b* parameter (obtained from fits of 1/*k_obs_* *vs.* Ф_I_ at constant [RES_T_], Ф_W_ and Ф_O_, Equation (9)) with the partition constant between corn oil and water in the absence of emulsifier, *P*_W_^O^, which is numerically equal to the ratio, *P*_W_^I^/*P*_O_^I^ (Equations (1)–(3)), and solving two equations with two unknowns. Once *P*_W_^I^ and *P*_O_^I^ are known, *k*_I_ can be determined by using Equation (7) [19].

## 2. Experimental Section

### 2.1. Materials

Resveratrol (Aldrich), caffeic acid (Aldrich), 2,2-diphenyl-1-picrylhydrazyl (DPPH^•^, Aldrich) stripped corn oil (Across Organics, ρ = 0.918 g/mL) and polyoxyethylene sorbitan monolaurate (Tween 20, Fluka) were of the highest purity available and used as received. Aqueous solutions were prepared by using Milli-Q-grade water. The acidity of the aqueous phase was controlled by employing a citric acid/citrate buffer (pH = 2, 0.04 M). Solutions of the coupling agent, *N*-(1-Naphthyl) ethylenediamine (NED, Aldrich), were prepared in a 50:50 (v/v) BuOH:EtOH mixture to give [NED] = 0.02 M. The chemical probe, 4-hexadecylbenzenediazonium tetrafluoroborate, 16-ArN_2_BF_4_, was prepared from commercial 4-hexadecylaniline (Aldrich, 97%) under nonaqueous conditions as described in a published method [21] and was stored in the dark at low temperature to minimize its decomposition. 

### 2.2. Emulsion Preparation

Emulsions 4:6 (v:v) were prepared by mixing 4 mL of stripped corn oil, 6 mL of citrate buffer (pH = 2, 0.04 M) containing 1.3 mM of RES and a weighed amount of non-ionic surfactant Tween 20 (Ф_I_ = *V*_surf_/*V*_emulsion_ = 0.005–0.04). The mixture was stirred with a high-speed rotor (Polytron PT 1600 E) for 1 min. The emulsions were then transferred to a continuously stirred thermostated cell, and their stability was checked visually; no phase separation was observed within 3–4 h, a time much longer than that required to complete the chemical reaction between 16-ArN_2_^+^ and RES.

### 2.3. Methods

#### 2.3.1. DPPH Radical Scavenging Method

Scavenging of the nitrogen centered radical, DPPH^•^, is the basis of a simple, rapid and commonly employed antiradical scavenging assay for evaluating the antioxidant activities of AOs in the micromolar range [22]. DPPH^•^ can react with phenols (ArOH) via a direct abstraction of phenol H-atom (HAT mechanism) or via an electron transfer process from ArOH or its phenoxide anion (ArO^−^) to DPPH^•^ (ET mechanism) [23]. The contribution of one or the other pathway depends on the nature of the solvent and/or the redox potentials of the involved species [24]. Generally, in apolar solvents, the HAT mechanism is predominant, but in polar solvents, such as methanol or ethanol, capable of forming strong hydrogen bonds with the ArOH molecules, the ET mechanism is predominant.

The DPPH radical absorbs at 517 nm, but upon reaction with an antioxidant, its absorption decreases. DPPH^•^ solutions were freshly prepared daily, stored in a flask covered with aluminum foil and kept in the dark at 4 °C between the measurements. Briefly, aliquots of RES in MeOH (50 µL, [RES] = 0.071–0.130 mM) were added to 250 µL of 0.096 mM DPPH^•^ in methanol. The absorbance of the resulting solution was measured by using a Powerwave XS Microplate Reader (Bio-Tec Instruments, Inc. (Carnaxide, Portugal). The microplate was thermostated at 25 ± 0.1 °C for all runs. The sample blank was prepared in the same manner, except that methanol (50 µL) was used instead of the aliquot of the RES stock solution (that is, 50 µL methanol plus 250 µL DPPH^•^ solution). All runs were carried out in quadruplicate, and the absorbance of each solution was recorded each minute for a 1-h period.

The EC_50_ values were determined as reported in the literature [24]. Briefly, the variation in DPPH^•^ absorbance with the time was plotted for several mol AO/mole DPPH^•^ ratios. The remaining percentage of the DPPH radical (%DPPH_rem_) was calculated as %DPPH_rem_ = [DPPH]*_t_*/[DPPH]*_t_*_ = 0_ at selected times with the aid of a previously prepared calibration curves. The obtained %DPPH_rem_ was then plotted against the mol AO/mol DPPH^•^ ratio for each selected time. The EC_50_ value was defined as the AO concentration that causes a decrease in the initial DPPH^•^ concentration of 50%.

#### 2.3.2. Oxidative Stability of Emulsions

The degree of oxidation of the emulsions was determined as in previous works [13,16] by monitoring the formation of conjugated dienes (CD) according to the AOCS Official Method Ti 1a-64. Briefly, stripped corn oil/Tween20/acidic water(buffered) emulsions were prepared, vortexed every 12 h for 60 s to prevent emulsion phase separation and allowed to oxidize spontaneously at T = 55 °C in the dark. Emulsions with no added antioxidant were used as the control. At selected times, 50 µL of each emulsion were diluted to 10 mL with ethanol, and the absorbance was determined at λ = 233 nm. All runs were performed in triplicate.

#### 2.3.3. Determining the Partition Constant, *P*_W_^O^, of Resveratrol in Binary Stripped Corn Oil-Water Mixtures in the Absence of Emulsifier

Determining *P*_W_^O^ is important to determining the distribution of RES in emulsions, because its value is numerically equal to the ratio of the partition constants between the oil-interfacial, *P*_O_^I^, and aqueous-interfacial, *P*_W_^I^, regions of emulsions, Equations (1)–(3). RES is a fat-soluble compound, which is also soluble in ethanol and dimethyl sulfoxide (DMSO). Its solubility in water is low, but not negligible (S = 0.13 mM) [8]. Its partition constant between *n*-octanol and water is known [8], logP = 3.1, but its partition constant between corn oil and water, *P*_W_^O^, has not been reported. 

**Figure 1:**
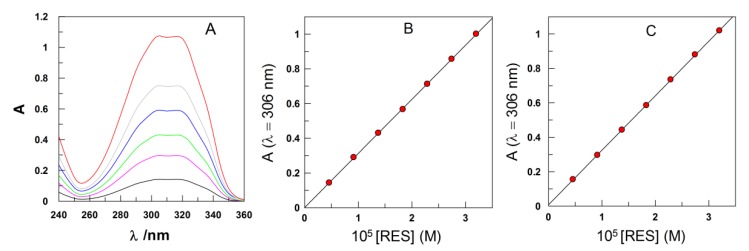
(**A**) Spectra of RES in water at different [RES]; (**B**) Beer’s law plot for RES in water (A = (7 ± 27) × 10^−4^ + (31,187 ± 130) [RES]); (**C**) calibration curve in a 50:50 (vol:vol) EtOH/BuOH mixture containing 40 µL of corn oil (A = (7.6 ± 2) × 10^−4^ + (31,682 ± 106) [RES]). Correlation coefficients > 0.999.

*P*_W_^O^ was determined by employing the same shake-flask method as employed in previous works [12,15,16]. RES was dissolved in 6 mL of a buffered (citric/citrate) aqueous acid (pH = 2.14) solution and mixed with 4 mL corn oil ([RES] = 4 × 10^−4^ M). The mixture was stirred at high speed for 1 min and allowed to reach thermal equilibrium for at least 4 h. The phases were then separated by centrifugation, and the concentrations of RES in the aqueous and oil phases were determined using UV spectrometry by interpolation in a previously prepared calibration curve. Figure 1A shows the spectra of RES in water at different [RES]. The calibration curve in Figure 1B was prepared in water by measuring the absorbance at increasing [RES], from where a value of ε_306_ = 31,187 ± 130 M^−1^cm^−1^ was obtained. The concentration of RES in oil was determined from the absorbance of a solution obtained by diluting an aliquot (40 µL) of the oil in a 50:50 (v:v) EtOH/BuOH mixture; Figure 1C. In all cases, the correlation coefficients were higher than 0.999. The partition constant, *P*_W_^O^, was determined in triplicate as the ratio of the concentrations of RES in the oil (%RES_O_) and water (%RES_W_) by employing Equation (1).

The average value of the determined percentage of RES in the aqueous phase was %RES_W_ = 74.6 and in the oil phase %RES_O_ = 23.8 (total recovery 98, 4%), and by employing Equation (1), an average value of *P*_W_^O^ = 4.7 ± 0.1 was determined. This *P*_W_^O^ value indicates that RES is about 5 times more soluble in oil than in water.

Partitioning of AOs (and solutes, in general) between different phases or regions within solutions depends on both the capability of the AO of intra-and inter-molecular hydrogen bonding with solvents and the differences in packing by solutes with solvent molecules or surfactants. This balance cannot be evaluated solely on the basis of their molecular structure. For instance, the solubilities in water of hydrophilic antioxidants, such as gallic acid, caffeic acid or hydroxytyrosol (which has the same number of hydroxyl groups in their structure, but only one aromatic ring), are relatively high [25,26], S = 10 g/L, S = 2.92 g/L and S = 50 g/L, respectively, and their *P*_W_^O^ values are low. As a consequence, the *P*_W_^O^ value obtained for RES in corn oil/water mixtures is about 100 times higher than those obtained for hydrophilic antioxidants, such as gallic acid (GA), caffeic acid (CA) or hydroxytyrosol (HT), *P*_W_^O^ = 0.03 [15], *P*_W_^O^ = 0.04 [14] and *P*_W_^O^ < 0.02 [16], respectively, indicating the higher solubility of RES in corn oil than CA, GA or HT.

It may also be worth noting that the determined *P*_W_^O^ value is ~300 times lower than the reported *p*-value in 1-octanol/water mixtures [8], log*P* = 3.1. This huge difference between *P* and *P*_W_^O^ indicates that RES must be much more soluble in 1-octanol than in corn oil and suggests that *P-*values cannot be employed to predict the hydrophobicity of AOs in edible oils.

#### 2.3.4. Determining *k*_obs_ Values in Emulsions: Derivatization Method

**Scheme 3:**
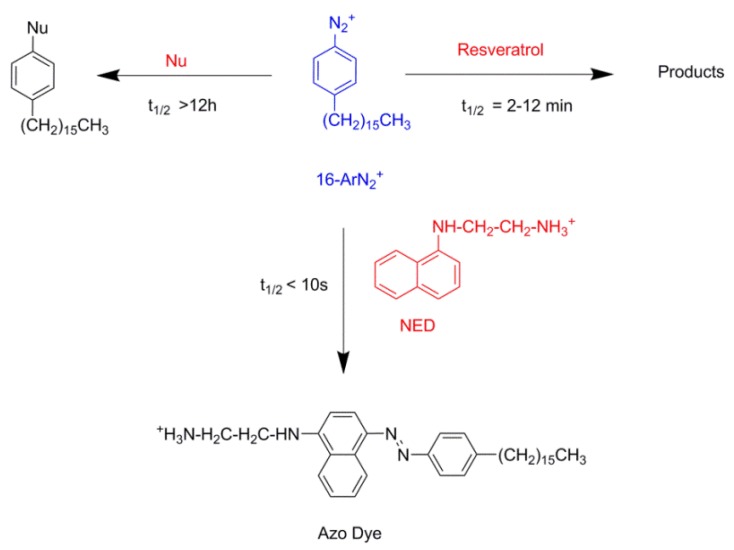
Values of the half-lives, *t*_1/2_, for the spontaneous decomposition of 16-ArN_2_^+^ and for the reactions with RES and *N*-(1-naphthyl)ethylenediamine (NED) obtained under the experimental conditions. Note that the reaction of 16-ArN_2_^+^ with NED is much faster than its spontaneous decomposition and than with RES, a requirement to get reliable rate constants by using the derivatization method [27].

**Figure 2:**
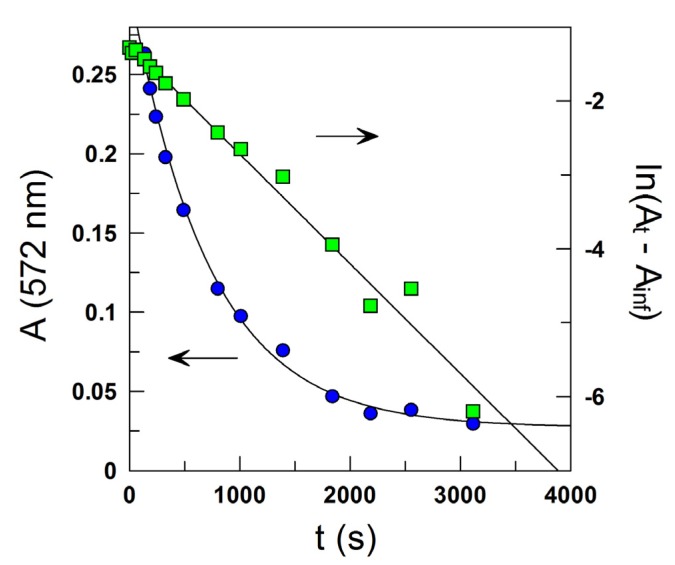
Typical kinetic plot illustrating the determination of *k*_obs_ for the reaction between 16-ArN_2_^+^ and RES in corn oil emulsions from the variation of the absorbance of the azo dye (λ = 572 nm) with time (-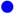-) and ln plot (-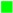-), according to Equation (10). Reaction conditions: Ф_I_ (Tween 20) = 0.0289, [16-ArN_2_^+^] = 1 × 10^−4^ M, [RES] = 1.3 mM, pH 2.14 (0.04 M citric-citrate buffer), T = 25 °C.

Because emulsions are opaque, classical spectroscopic methods to determine rate constants in homogeneous solution, such as UV-Vis, cannot be used. We developed a special protocol, based on the derivatization of the chemical probe, 4-hexadecylbenzenediazonium, 16-ArN_2_^+^, with the coupling agent, *N*-(1-naphthyl)ethylenediamine, NED, Scheme 3 [20,27]. The formed azo dye was diluted with a 50:50 (v:v) BuOH:EtOH mixture to yield an optically transparent solution, whose absorbance is measured spectrometrically at λ = 572 nm. Experimental conditions were optimized, so that the reaction of 16-ArN_2_^+^ with NED (*t*_1/2_ < 10 s) [27] is much faster than the spontaneous decomposition of 16-ArN_2_^+^ (*t*_1/2_ > 12 h) with RES (*t*_1/2_ 2–12 min), Scheme 3. Reactions were carried out under pseudo-first order conditions, being [RES] much higher than [16-ArN_2_^+^], and monitored for at least 2–3 *t*_1/2_. *k*_obs_ values were obtained by fitting the absorbance-time pairs of data to the integrated first order Equation (10) (Figure 2), using a non-linear least squares method provided by a commercial computer program (GraFit 5.0.5). In Equation (10), *A*_t_, *A*_o_ and *A*_inf_ are the measured absorbance at any time, at *t* = 0 and at infinite time.


1n(A_t_ - A_inf_)= -*k*_obs_t + 1n(A_0_ - A_inf_) (10)

Typical correlation coefficients were >0.99 in all runs, and duplicate or triplicate experiments gave *k*_obs_ values with deviations lower than 7%. Auxiliary experiments show that size of the emulsion droplets and their variation with the time does not affect kinetic results.

## 3. Results and Discussion

### 3.1. Distribution of RES in Stripped Corn Oil Emulsions

**Figure 3:**
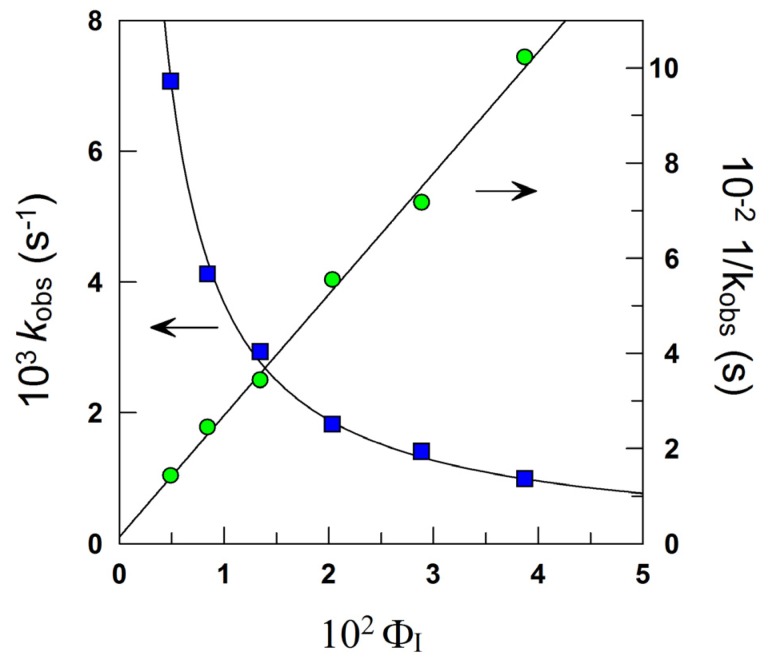
Effects of increasing Ф_I_ on *k*_obs_ for the reaction of 16-ArN_2_^+^ with resveratrol in 4:6 stripped corn oil/Tween 20/acidic water emulsions. Experimental conditions: [16-ArN_2_^+^] = 1.034 × 10^−4^ M, [RES] = 1.3 mM, pH 2.14 (citrate buffer 0.04 M), [NED] 0.02 M, T = 25 °C.

Figure 3 shows the variation of *k*_obs_ and 1/*k*_obs_ with Ф_I_ in 4:6 emulsions composed of stripped corn oil and acidic water (buffered, pH = 2.14). Values of *k*_obs_ decrease by a factor of ~7 on going from Ф_I_ = 0.005 to Ф_I_ = 0.04 in keeping with the predictions of Equation (6). The decrease of *k*_obs_ with Ф_I_ is similar to those found for caffeic acid [14] (~6) and catechin [28] (~6), but somewhat higher than those found for antioxidants, such as gallic acid [15] (~4), catechol (~3) and hydroxytyrosol [16] (~3).

The solid lines in Figure 3 are the theoretical curves obtained by fitting the experimental data to Equations (6) and (9), and the excellent fits obtained indicate that the assumptions of the pseudophase model are fulfilled. By employing Equations (7) and (8) (obtained by fitting the (1/*k*_obs_, Ф_I_) pairs of data to Equation (9)) with the determined *P*_o_^W^ value, values of *P*_W_^I^ = 4374, *P*_O_^I^ = 930 and *k_I_* = 3.02 × 10^−2^ M^−1^ s^−1^ were determined. The *k_I_* value obtained is similar to the reported value for caffeic acid (*k_I_* = 3.64 × 10^−2^ M^−1^ s^−1^), but lower than those for catechin (*k_I_* = 6.50 × 10^−2^ M^−1^ s^−1^) or catechol (*k_I_* = 7.65 × 10^−2^ M^−1^ s^−1^) in corn oil emulsions.

*k_I_* reflects the medium effects on *k*_obs_. Its values are not necessary for the assessment of the antioxidant distributions; however, they are valuable, because comparison of *k*_I_ values for a number of AOs could be used as a basis for assessing a scale of AO activity that is independent of the AO distribution in the emulsion, because the same chemical probe (16-ArN_2_^+^) is used in all distribution experiments [19,20]. Analyses of their values for series of AOs can afford insights into the reaction mechanism in the interfacial region of emulsions [13,14]. Changes in *k_I_* values may also detect changes in the reaction mechanism [19]. For instance, the variation of *k_I_* with the acidity allows the determination of whether the reactive species is the anionic, dianionic or neutral form of the AO. Moreover, estimations of the activation parameters for the reaction with 16-ArN_2_^+^ in emulsions can be obtained from the variations of *k_I_* with temperature.

The partition constant values *P*_W_^I^ = 4374 and *P*_O_^I^ = 930 are high compared to the reported values for hydrophilic (*P*_W_^I^ = 250 for caffeic acid [14], *P*_W_^I^ = 119 for gallic acid [13], *P*_W_^I^ = 370 for catechin [28]) and hydrophobic AOs, such as tocopherol [29], *P*_O_^I^ = 11, or lauryl gallate [13], *P*_O_^I^ = 19. Substantial *P*_W_^I^ values as that found here suggest the high tendency of RES to be incorporated into the interfacial region of emulsions. Nevertheless, an increase in *P*_W_^I^ values does not necessarily imply increases in *P*_O_^I^ values in spite of the increase in the hydrophobicity of the AO, because they can be dissolved in the oil region, as we recently found for a series of gallic [13] and caffeic [30] acid derivatives of increasing hydrophobicity. High *P*_W_^I^ and *P*_O_^I^ values indicate, in general, the tendency of the AOs to be located exclusively in the interfacial region of the emulsion, as we found for gallic acid derivatives in octane-based emulsions [19].

**Figure 4:**
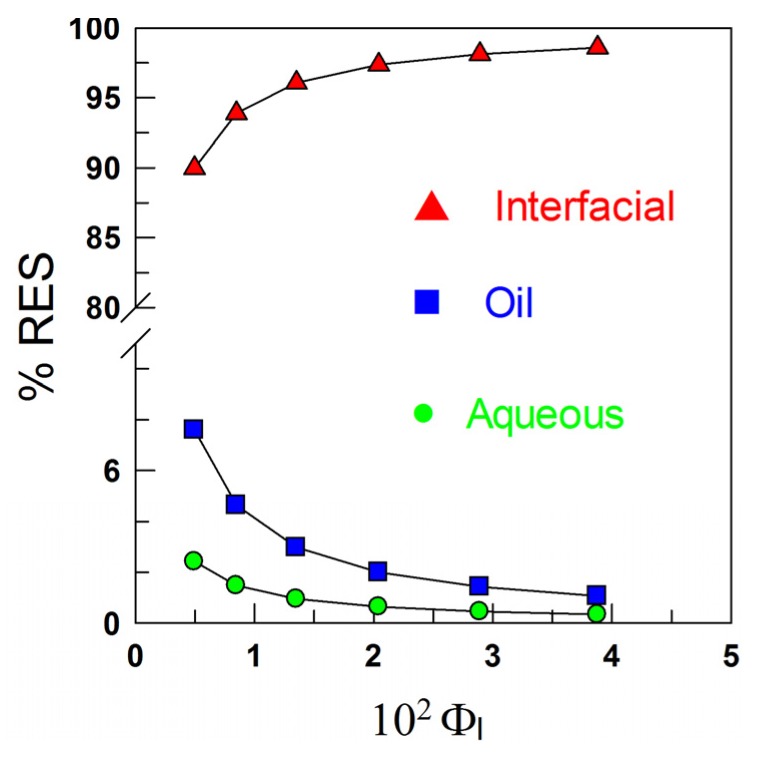
Distribution of resveratrol in a 4:6 (O/W) corn oil/Tween 20/acidic water emulsion.

Once the partition constants, *P*_W_^I^ and *P*_O_^I^, are known, the determination of the percentage of the resveratrol in the oil, aqueous and interfacial regions is straightforward by employing Equations (11)–(13), which have been derived by combining the expressions of partition constants and the mass balance equations. Details can be found elsewhere [19]. Figure 4 shows the distribution of RES in corn oil emulsions as a function of the emulsifier volume fraction. RES is primarily located in the interfacial region of the corn oil emulsions, %RES_I_ = 90% at Ф_I_ = 0.005, and this percentage increases to %RES_I_ = 99% at Ф_I_ = 0.04. That is, RES is primarily located in the interfacial region of the emulsion at the lowest Ф_I_, and its percentage only increases ~10% upon increasing the interfacial volume eight-fold. 


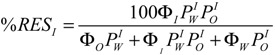
(11)


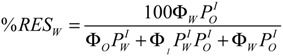
(12)


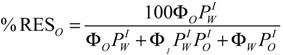
(13)

### 3.2. Oxidative Stability of Emulsions

**Figure 5:**
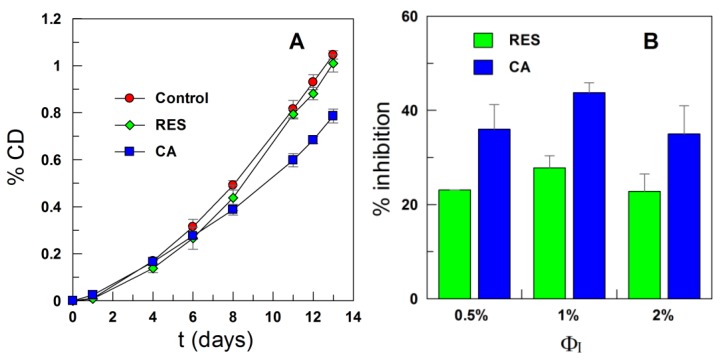
(**A**) Oxidative stability of stripped corn oil in water emulsions (4:6) obtained in the presence of RES and caffeic acid (CA) as determined by the change in the formation of conjugated dienes with the time at T = 55°C; and (**B**) inhibition by RES and CA on the formation of conjugated dienes in stripped corn oil emulsions during oxidation at T = 55 °C. Values were taken on Day 13. The percentage of inhibition was calculated as % Inhibition = [(C − S)/C] × 100, where C is the increment in the %CD (conjugated dienes) of the control and S is the increment in the %CD of emulsions in the presence of antioxidant (AO; [AO] = 0.6 mM in the corn oil and F_Tween 20_ = 0.01).

Stripped corn oil emulsions with different emulsifier concentrations (Ф_I_ = 0.005, 0.01 and 0.02) were prepared in the presence and absence of RES, and their degree of oxidation was monitored at T = 55 °C by measuring the conjugated dienes content; Figure 5.

**Figure 6:**
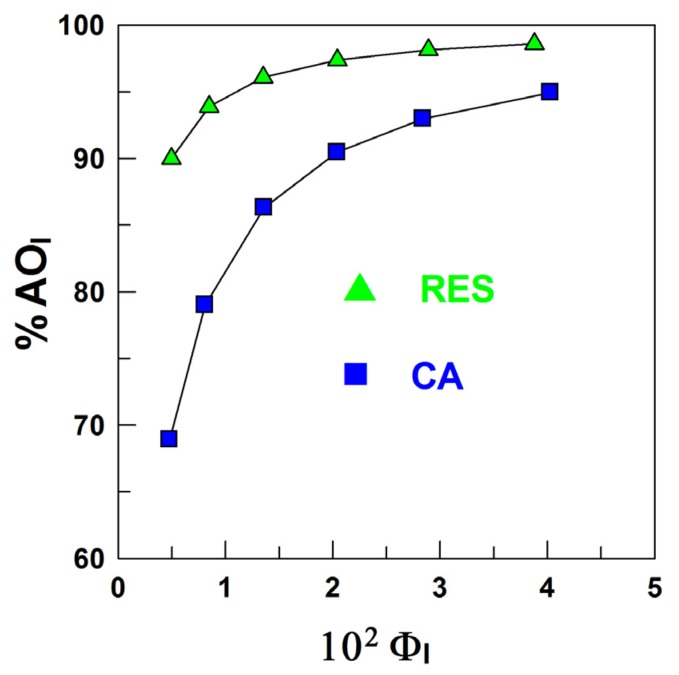
Percentage of resveratrol (-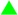-) and caffeic acid (-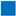-) in the interfacial region of corn oil in water emulsion (4:6, O/W). Experimental conditions: [RES] = 1.3 mM, [CA] = 4.4 mM, citrate buffer 0.04 M (pH 2.14 for RES and pH 3.65 for CA). T = 25 °C. Data for CA were taken from [30].

As shown in Figure 5A, the effect of RES in inhibiting the oxidation of emulsified corn oil lipids is negligible. The results may be surprising, because the distribution experiments show that more than 90% of RES is located in the interfacial region of the emulsions (Figure 4), and we demonstrated in recent work [13,30] that for a series of gallate and caffeate alkyl esters, the AO with the highest percentage in the interfacial region (the butyl and octyl ester respectively) is the one which shows the highest antioxidant efficiency. To rationalize this observation and for comparative purposes, we investigated the efficiency of caffeic acid, because we previously determined its distribution in emulsions of the same composition [30]. Figure 5 shows that CA is more efficient than RES and appreciably inhibits the oxidation of the emulsions at any selected emulsifier volume fraction, in spite of the much lower fraction of CA in the interfacial region of emulsions when compared with that of RES (Figure 6).

The efficiency of AOs depends not only on the rates of the chemical reactions involved, but also on reactant concentrations in the interfacial region, currently believed to be the primary site of oxidation [31,32]. Literature reports indicate that the efficiency of phenolic AOs depends on the number and position of hydroxyl groups in their structure [1,31,33]. AOs with –OH groups in the *ortho* positions, such as catechol derivatives, are powerful antioxidants, because of their low oxidation potentials and their ability to stabilize the semiquinone radicals derived from H-atom donation of catechol by an intramolecular hydrogen bond and the electron-donating properties of the *ortho*-OH [34]. Therefore, the negligible efficiency of RES may be a consequence of the lower reactivity of RES towards the radicals than phenolic antioxidants, such as caffeic acid. To test this hypothesis, we determined, as in previous works [13,30], their radical scavenging activity by employing the DPPH method in homogeneous solution to avoid the concentration effects inherent to systems with regions of different solvent properties (e.g., emulsions).

**Figure 7:**
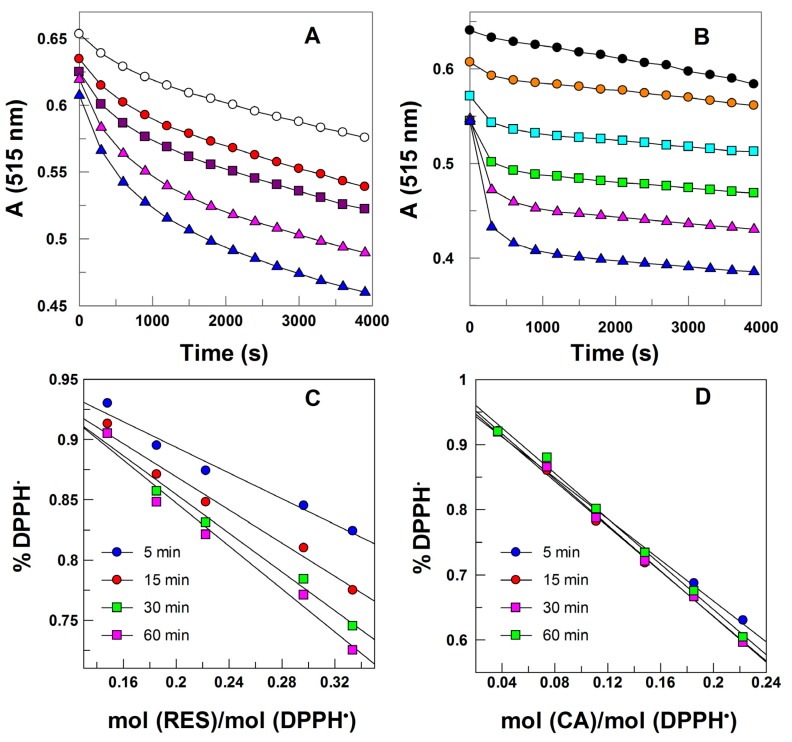
(**A**) Variation of the absorbance of 2,2-diphenyl-1-picrylhydrazyl (DPPH) in the presence of different concentrations of RES at T = 25 °C (-Ο- 0.1483 mol RES/mol DPPH, -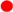
- 0.1854 mol RES/mol DPPH -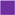
- 0.2223 mol RES/mol DPPH, -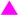
- 0.2966 mol RES/mol DPPH, -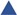
- 0.3337 mol RES/mol DPPH); (**B**) variation of the absorbance of DPPH in the presence of different concentrations of CA at T = 25 °C (-●- 0.0371 mol RES/mol DPPH, -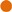
- 0.0742 mol RES/mol DPPH, -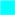
- 0.1113 mol RES/mol DPPH, -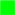
- 0.1484 mol RES/mol DPPH, -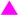
- 0.1855 mol RES/mol DPPH, -▲- 0.2226 mol RES/mol DPPH). (**C**,**D**) Determination of EC50 values of RES; (**C**) and CA; (**D**) towards DPPH free radicals.

Figure 7A,B show the variation in DPPH absorbance with the time at different mol AO/mol DPPH ratios for RES and CA, respectively. Figure 7C,D was prepared as indicated in Section 2.3.1, and the corresponding EC_50_ values were determined at selected times (Table 1).

The EC_50_ value for RES at 5 min is almost three times higher than that for CA, indicating a much higher reactivity of CA towards DPPH^•^ compared to that of RES. This difference in reactivity is in keeping with the reported [24] ratio of the rate constants for the reaction of DPPH^•^ with CA (*k* = 539 M^−1^ s^−1^) and RES (*k* = 138 M^−1^ s^−1^). The higher radical scavenging activity of CA compared to that of RES is probably a consequence of the presence of the hydroxyl group in the *ortho* position, which facilitates the formation of stable quinones in spite of the delocation of the electrons over all the RES molecule.

**Table 1:** EC_50_ values in moles of antioxidant (resveratrol and caffeic acid) per mol of DPPH obtained at reaction times of 5, 15, 30 and 60 min.

Antioxidant	*t* = 5 min	*t* = 15 min	*t* = 30 min	*t* = 60 min
RES	0.9080	0.6991	0.5991	0.5256
CA	0.3016	0.2791	0.2775	0.2843

## 4. Conclusions

We have determined the partition constants, *P*_W_^I^ and *P*_O_^I^, and the distribution of RES in stripped corn oil emulsions. RES is primarily located in the interfacial region, %RES_I_ > 90% at the lowest emulsifier volume fraction, and %RES_I_ increases by only 10% on increasing the surfactant volume fraction 100-fold, indicating that %RES_I_ is essentially independent of Ф_I_. It is worth noting that, at any given Ф_I_, %RES_I_ is much higher than the percentages of both hydrophobic AOs, such as tocopherol, alkyl gallates and alkyl caffeates and hydrophilic AOs, such as gallic and caffeic acids, catechin and hydroxytyrosol, confirming that any prediction of the percentage of AOs in the interfacial region of emulsions on the basis of their hydrophobicity is unreliable, because %AO_I_ does not correlate with AO hydrophobicity, as we previously reported [13,30].

Our results show that RES is not efficient in inhibiting the oxidation of the emulsified corn oil lipids in spite of the high percentage of RES in the interfacial region, in contrast with results obtained for caffeic acid, whose efficiency is much higher than that of RES, but whose percentage in the interfacial region is much lower. DPPH experiments showed that the reactivity of RES towards the DPPH radical is much lower than that of caffeic acid. Thus, our results indicate that to assess the efficiency of AOs in emulsified systems, both their concentration at the reaction site and their reactivity towards the radicals needs to be considered. Moreover, our results predict that an increase in the hydrophobicity of RES by lipophilization will not result in an improvement of its efficiency, because most RES is located in the interfacial region of the emulsions, as Medina *et al.* [10] have demonstrated experimentally. 

Our results also show that the kinetic method employed to determine AOs distributions in the intact emulsions is robust, feasible and provides reliable results. No other method is currently available. Establishing relationships between antioxidant efficiency and antioxidant partitioning is basic to selecting the best set of antioxidants for a given food system and, so, to prepare healthier and more nutritional foods with a longer shelf-life. The results obtained here contribute to understanding how the hydrophobicity of AOs controls their distribution in emulsified systems, which is basic to understanding their effectiveness in inhibiting lipid oxidation. However, partitioning of AOs depends on a number of parameters and further investigations on the role of these parameters (temperature, acidity, hydrophilic-lipophilic balance (HLB) of the antioxidant and emulsifier, nature of the oil, and so forth) are in progress and will be part of future reports.
